# Insights Into Self‐Care of Feet When Living With Diabetes: A Phenomenological Hermeneutic Interview Study

**DOI:** 10.1111/jocn.70346

**Published:** 2026-05-07

**Authors:** Kristofer Björk, Henrik Eriksson, Ulla Hellstrand Tang, Susanne Andersson

**Affiliations:** ^1^ University West Department of Health Sciences Trollhättan Sweden; ^2^ Department of Prosthetics & Orthotics Sahlgrenska University Hospital Gothenburg Sweden; ^3^ Department of Orthopaedics, Institution of Clinical Sciences, Sahlgrenska Academy University of Gothenburg Mölndal Sweden; ^4^ Research & Develeopment Centre Skaraborg Skövde Sweden

**Keywords:** diabetes, diabetic foot, learning, lived experience, nursing, phenomenological hermeneutic, qualitative research, self‐care

## Abstract

**Aim:**

To describe how persons with diabetes experience learning about self‐care, with a particular focus on foot self‐care.

**Methods:**

Narrative, semi‐structured interviews were conducted with persons diagnosed with Type 1 or Type 2 diabetes. Participants were recruited through an advertisement in a diabetes association magazine and through convenience and snowball sampling. Eleven participants aged 53–87 were interviewed between December 2024 and April 2025. Interviews were audio‐recorded, transcribed verbatim and analysed using a phenomenological hermeneutic approach.

**Results:**

Learning about foot self‐care emerged as a gradual, lived process shaped by bodily experiences, social relationships and access to support. Three themes were identified: the social landscape of self‐care, the being of the feet and taking knowledge into your own hands. Learning was influenced by encounters with healthcare professionals, informal support from relatives and personal experiences over time. Bodily symptoms acted as driving forces for learning and self‐care actions. Understanding why self‐care mattered was essential for motivation, while seeking and evaluating knowledge became a strategy for control and participation in care.

**Conclusion:**

Learning about foot self‐care among persons with diabetes is an embodied, relational and ongoing process. That develops through interaction among lived bodily experiences, social support and personal responsibility rather than through information alone.

**Implications for the Profession and/or Patient Care:**

Healthcare professionals must integrate persons' experience‐based knowledge and support dialogue, reflection and shared learning to strengthen foot self‐care practices.

**Impact:**

The study addressed the limited understanding of how persons with diabetes learn foot self‐care. The results showed that learning is shaped by lived experience, relationships and meaning‐making. The findings are relevant for persons with diabetes and healthcare professionals involved in diabetes care.

**Reporting Method:**

The study adhered to the COREQ criteria.

**Patient or Public Contribution:**

No patient or public contribution.

## What Does This Paper Contribute to the Wider Global Clinical Community?

It reframes foot self‐care learning as an existential, lifeworld‐oriented process rather than a series of clinical and technical routines.

It introduces phenomenological hermeneutics as a method for interpreting the essential meanings in individuals' narratives, making it possible to reveal person‐centred learning approaches.

It challenges narrow assumptions by showing that learning (of foot self‐care) is shaped by bodily signals, social relationships and personal agency, which necessitates flexible, dialogical (learning) approaches beyond standardised (frameworks) of educational protocols.

## 1 Introduction

There is a general lack of understanding about the important role of the feet and the biomechanical complexity of the foot and its crucial role in mobility and balance (Ingold 2004), which also extends to person's understanding of self‐care of the feet. This lack of recognition also extends to understanding of foot self‐care. Among persons with diabetes, the importance of foot self‐care may be limited and lead to delayed care seeking, which increases the risk of serious complications (McPherson et al. 2022). International guidelines encourage persons with diabetes to practice self‐care, which includes inspecting their feet daily to identify whether any signs of foot problems have arisen, washing the feet daily, drying between toes, taking care of the nails, using emollients, checking footwear and, if the person has neuropathy, using appropriate footwear both outdoors and indoors, using seamless socks, and protecting the feet from heat and cold (International Working Group on the Diabetic Foot 2023). To further understand the significance of the feet, lifeworld theory offers a valuable framework that emphasises how persons access the world through their lived bodies (Heidegger 1927/1967; Husserl 1907/1999; Merleau‐Ponty 1945/2002; Van Manen 1997), which provides valuable insight into the lived experiences of persons with long‐term illness, such as diabetes (Kjellsdotter et al. 2020). We have used the term ‘persons with diabetes’ because each person with the disease is unique, which aligns with the principles of person‐centred care (McCormack and McCance 2017).

The lived body is how we experience the world through our bodies (Van Manen 1997). In the context of living with diabetes, the lived body is significantly affected by symptoms such as fatigue, fluctuations in blood sugar levels (Johansson et al. 2016), and potential complications such as neuropathy (Eid et al. 2023). These physical challenges can potentially lead persons with diabetes to feel vulnerable, frustrated or self‐conscious about their appearance and identity (Vanstone et al. 2015). They can also affect how these persons perceive themselves and are perceived by others (Hosono and Tochikawa 2022; Johansson et al. 2016).

Lived time is the subjective experience of time (Van Manen 1997). Persons living with diabetes may experience a sense of uncertainty about the future. The constant need to keep the disease under control can create a feeling of being trapped in the present, with limited room for spontaneity. This can affect how persons with diabetes experience and plan for their future (Morales‐Brown et al. 2024).

The concept of lived space is intimately linked to a person's sense of security and freedom of movement (Van Manen 1997). Feeling safe when you have diabetes may mean having access to food and medication. This may mean that a fear of hypoglycaemic episodes can be limiting and affect a person's independence and mobility (Johansson et al. 2016).

In the context of a long‐term illness, such as diabetes, the person's learning and ability to self‐care becomes a central part of their daily life (Berglund 2014). Studies have shown that persons with other long‐term illnesses, such as Parkinson's disease and multiple sclerosis, learn to live with their illness based on their changing body and the physical changes it brings (Haahr et al. 2011; Strickland et al. 2017). Learning to live with a long‐term illness requires both self‐reflection and reflection together with others who have the same illness, confronting fears, recognising one's vulnerability and daring to make decisions leads to a more authentic and conscious life, which contributes to maintaining a sense of normality (Berglund and Källerwald 2012; Berglund 2014; Kjellsdotter et al. 2020). Others have described learning as taking control and protecting the body from harm, in both the short and long term. Learning involves integrating diabetes into the person's self‐understanding, enabling him or her to live a life under changed conditions, with a sense of responsibility for health and fear of future complications (Hosono and Tochikawa 2022; Johansson et al. 2016).

Focusing on practical, task‐oriented learning, supported by regular feedback, means that persons with diabetes are better equipped to manage their self‐care. Incorporating integrative and reflective learning methods further strengthens these outcomes. These educational processes help develop the skills needed to manage foot self‐care and prevent foot complications (Björk et al. 2025). Additionally, four key health behaviours have been identified for persons at risk of developing foot complications: controlling blood glucose levels, attending annual foot screenings, promptly reporting any changes in foot health and maintaining a daily foot self‐care routine (McInnes et al. 2011). Barriers to learning about foot self‐care can include geographical, administrative and communication challenges and limited access to specialists. Strategies like structured foot care programmes, educational initiatives and role definitions for healthcare professionals can enhance patient learning. By addressing these barriers, healthcare providers can improve patients' understanding of self‐care of the feet (Mullan et al. 2021). A structured form for foot examination strengthened patients' self‐care as the conversation focused on preventive foot health. The form also clarified the criteria for foot care for both patients and other healthcare providers, ensuring that referrals were justified (Andersson et al. 2023).

For persons with diabetes, self‐care is a constant part of daily life. However, there is insufficient understanding of the importance of foot self‐care and related complications. Additional challenges create the need for support in collaboration with healthcare providers. Learning processes vary from person to person, and healthcare professionals must consider different needs and learning styles. Needs and abilities are the basis for learning self‐care. Lifeworld theory provides a framework for an increased understanding of how persons with long‐term illnesses may experience and cope with living with diabetes. There is a need to understand the multi‐facetted manner in which persons with diabetes learn about self‐care of their feet.

## 2 Aim

The aim of this study was to describe how persons with diabetes experience learning about self‐care, with a particular focus on foot self‐care.

## 3 Method

We chose Lindseth and Norberg's (2004) phenomenological hermeneutic method for this study. The essential meaning of the phenomenon was explored and described as it emerged. The Consolidated Criteria for Reporting Qualitative Research (COREQ) checklist was used to ensure rigorous reporting of methods and results (Tong et al. 2007).

### 3.1 Data Collection

The inclusion criteria were being 18 years or above and diagnosed with either Type 1 or Type 2 diabetes. Exclusion criteria were impaired cognitive function and an inability to speak or understand Swedish. After the first pilot interview, included in the study, recruitment was initiated through an advertisement published in a magazine of the Diabetes Association in south Sweden. Eligible participants who responded and met the inclusion criteria were included in the study. Thereafter, a combination of convenience sampling and snowball sampling was used, where participants were asked to recommend other potential study participants. This combination of recruitment strategies enabled access to broad group of participants (see Table 1).

**TABLE 1:** Participant characteristics.

Participant	Sex	Age	Diabetes type	Years of diabetes
1	Male	73	Type 1	39
2	Male	77	Type 2	25
3	Female	80	Type 2	35
4	Female	70	Type 1	40
5	Male	73	Type 1	30
6	Female	80	Type 1	65
7	Male	74	Type 1	24
8	Male	60	Type 2	1.5
9	Male	54	Type 2	1.5
10	Female	63	Type 1	59
11	Female	87	Type 2	38

The author (KB), a registered nurse with experience in patient interactions, conducted the interviews between December 2024 and April 2025 in settings that the participants chose to create a comfortable atmosphere. These settings included workplaces, homes, country houses, participants' cars or healthcare clinics. The interviewer withheld personal and professional background information prior to the interviews to minimise influence, such information was shared only after data collection. After informant consent, the interviews began with informal small talk to create a respectful and friendly atmosphere. The interviews were semi‐structured and included follow‐up questions such as ‘How do you learn?’ and ‘What is your experience?’. The interviews were digitally recorded with accompanying notes, lasted between 21 and 57 min, and were transcribed verbatim.

### 3.2 Data Analysis

The interviewer (KB) listened and transcribed all the interviews to ensure accuracy and familiarity with the data. The analysis process included three distinct phases: naïve reading, structured analysis and comprehensive understanding (Lindseth and Norberg 2004).

In the first phase, the transcribed interviews were read in their entirety to gain a naïve understanding of the participants' experiences (KB). The initial step provided a first overall understanding of the interviews to understand the data on an intuitive level.

In the second phase, structural analysis was initiated and two authors (KB and SA) identified meaning units from the transcriptions. During this process, the text was decontextualised to focus solely on the units of meaning and to organise the units regardless of their original context in the text. Meaning units were then identified and condensed while retaining the essential meaning. These condensed meaning units were compared for similarities and differences, with similar units grouped together to form subthemes, and further abstracted into themes. The initial grouping was then completed. All authors actively contributed to the further refinement and development of these subthemes and themes (see Table 2). All authors were also involved in critical reflection on the subthemes and themes and consideration of their relationship to the naïve understanding.

Finally, a comprehensive understanding was reached that considers both the naïve understanding and the validated themes grounded in a broader theoretical context by all authors.

### 3.3 Ethical Considerations

Ethical considerations adhered to the research ethics guidelines outlined in the Declaration of Helsinki (World Medical Association 2024). Ethical approval from the Swedish Ethical Review Authority (Dnr. 2024‐07221‐01) was granted on 26 November 2024.

## 4 Findings

### 4.1 Naïve Understanding

Learning occurs gradually when the need for new knowledge becomes evident and not everything is recognised at once. Foot self‐care often takes a less prominent place initially but becomes increasingly important over time. Participants described that foot self‐care requires both precision and perseverance, as well as different forms of support. While healthcare professionals can provide a sense of security, that is not always enough. This means that persons with diabetes learn a lot through personal experience or get help from relatives. Thus, foot self‐care does not become an isolated activity, but rather an integral part of long‐term self‐care, shaped by bodily changes over time and by the available resources, social relations and contextual conditions surrounding the person.

### 4.2 Structured Analysis

**TABLE 2:** Themes and subthemes identified in analysis.

Themes	Subthemes
The social landscape of self‐care	Sense of security and insecurity in relation to care encounter
	Professional support – not just information and treatment
	Informal support and learning outside healthcare
The being of the feet	Self‐care ability – between habit, will and obstacles
	Learning through experience, education and understanding
	Bodily symptoms as a driving force for action
Taking knowledge into your own hands	Seeking knowledge as a strategy for control and understanding
	Understanding as a prerequisite for motivation
	Trust in science and a critical attitude to new knowledge

#### 4.2.1 The Social Landscape of Self‐Care

Learning about self‐care of feet in diabetes occurs primarily through interaction with healthcare professionals and informal networks around the person. It is shaped in relation to others, within a social landscape that encompasses a non‐linear learning process reinforced through relationships and experiences. When healthcare is perceived as accessible and engaged, both knowledge and motivation for self‐care are strengthened. When family members are involved, self‐care of the feet becomes a shared responsibility. At the same time, a lack of support or prioritisation can create uncertainty and limit opportunities for learning. Thus, the social landscape of self‐care means that learning is formed together with others.

##### 4.2.1.1 Sense of Security and Insecurity in Relation to Care Encounter

Meeting with healthcare professionals is a crucial factor in feeling confident regarding self‐care of the foot. It is not only about receiving advice or treatment about self‐care, but also about trusting that help is available and taken seriously when needed. Being cared for creates feelings of trust and security. As one participant put it: ‘If any problems arise, I know who to contact, and I will get help fairly quickly, so I feel secure’ (D1). Receiving feedback on one's self‐care and being aware of changes gives person with diabetes a feeling of being seen and supported.

This sense of security can be replaced by insecurity. When access to professional foot care is limited, or when participants are perceived as ‘too healthy’, they may feel devalued. This sense of security is quickly affected.

##### 4.2.1.2 Professional Support—Not Just Information and Treatment

Professional support is shaped by relationships with different healthcare professionals and by how attentive and engaged the professionals are. This support includes practical examinations such as foot examinations and advice on self‐care, including considering the person's entire life situation. A sense of belonging arises when professional contacts are continuous and based on genuine interest. One participant illustrated this point, as follows: ‘But I have to add that I have a wonderfully skilled diabetes nurse. Not as skilled doctor. But the diabetes nurse is so kind. She calls, follows up, adjusts, regulates, and collaborates all the time … The doctor, on the other hand, is … stupid. And I say that because my husband had kidney problems, and the doctor didn't take it seriously’ (D11).

When the person is listened to during counselling, a sense of trust develops. A lack of responsiveness can create distance and, in some cases, lead to distrust and frustration.

##### 4.2.1.3 Informal Support and Learning Outside Healthcare

When learning about foot self‐care family members also play a central role by providing knowledge, practical care and reminders of routines. The informal support from the family offers a sense of security that both facilitates self‐care and strengthens the person's ability to manage the condition. One participant described it as follows: ‘I used to get foot care from … the foot care specialist … we didn't quite get along, I think. So now my husband is my foot care specialist, and he takes care of my feet – and they've never looked this good’ (D4). As aging or reduced mobility make foot care more difficult, the partner's help becomes particularly important.

Self‐care of the feet can be learned in many contexts, such as patient associations, educational events or by sharing experiences with others in the same situation.

#### 4.2.2 The Being of the Feet

Being ‘in your feet’ means that your feet become a place where your body's signals remind you of vulnerability and the need to be mindful. Consequently, bodily symptoms become a central driving force that evokes concern while also prompting action and the adoption of new habits. Learning about self‐care is an ongoing process in which experiences, advice from professionals and reflections within one's social environment contribute to a deepened understanding. Thus, the feet become a source of knowledge, where the body itself signals what requires attention, and caring for one's feet becomes a way to preserve both health and quality of life. Therefore, self‐care ability is shaped within the tension between will and obstacles, between routines and resistance, and between limited professional care and the person's own responsibility that accompanies the illness. The being of the feet represents an embodied form of learning where experience, care and concerns are intertwined in a continuous negotiation of health, safety and quality of life.

##### 4.2.2.1 Self‐Care Ability—Between Habit, Will and Obstacles

Foot self‐care has been described as being characterised by established routines, opportunities, challenges and varying levels of motivation that sometimes make it difficult to follow recommendations. A strong sense of responsibility makes self‐care an integral part of everyday life to keep feet soft, prevent ulcers and maintain blood circulation. One participant expressed this as follows: ‘There's very little time devoted to a person with diabetes in healthcare. Maybe two or three hours a year … while I myself have it 24 seven’ (D1).

Routines such as practicing foot exercises are deeply rooted in previous experiences. One participant explained: ‘I've learned all sorts of foot exercises … Yes, we always did foot exercises in dance training to strengthen the muscles – spreading the toes, flexing the feet, placing a towel under the foot to curl it with your toes and then stretch out again’ (D10).

Some participants emphasised that self‐care can be difficult. Reaching the feet, cutting nails and moisturising the skin were mentioned as challenging, and self‐care routines can be time‐consuming and less important when other aspects of the disease require more attention. Neuropsychiatric difficulties may also complicate the establishment of consistent routines.

A strong desire to protect the feet emerges as a key motivation. Personal strategies include performing foot exercises, massage, using specific creams or using adapted footwear.

##### 4.2.2.2 Learning Through Experience, Education and Understanding

Learning about self‐care of feet develops gradually through a combination of experience, education and reflection with others. Combining advice from healthcare professionals, reading and personal experience can increase understanding and learning about foot self‐care. Structured education on self‐care of the feet is lacking and is not as prioritised as nutritional advice, which means that participants are largely left to learn on their own and rely on their own experiences. One participant described workplace learning as follows: ‘There were many who had problems with their feet and legs where I worked, and they received care and advice and all those things – and you listened and learned there and then’ (D4).

Learning is built progressively through different encounters with healthcare, through reading, asking questions and experimenting in daily life.

##### 4.2.2.3 Bodily Symptoms as a Driving Force for Action

Physical symptoms act as a driving force to seek care, ask questions, change routines and reinforce self‐care as something important. When numbness, pain or visible changes appear in the feet, the connection between the illness and the body becomes tangible, generating awareness of the need for action. As one participant described: ‘I can say my toes are ice cold almost every day. It feels like the circulation stops at the toes. It doesn't go up into the leg where I have more warmth – just the toes are ice cold’ (D3).

Bodily signals evoke concern and also function as a catalyst for action. Symptoms may lead to more frequent healthcare contacts or adjustments in daily routines, such as moisturising or changing shoes more often.

Despite the important impact of symptoms on self‐care efforts, professionals do not always prioritise feet. Other diseases that often occur simultaneously may be considered more important and urgent to manage, even if symptoms increase awareness and motivation to engage in self‐care.

#### 4.2.3 Taking Knowledge Into Your Own Hands

Learning about foot self‐care in diabetes does not take place solely within professional encounters. The outcome is shaped by the person's ability to seek knowledge and understand it in relation to their own life circumstances while living with the disease. Learning becomes more than a transfer of information. By searching for knowledge and asking questions that are evaluated, an understanding of one's own situation is created, which is translated into practical actions and leads to learning. Taking knowledge into one's own hands becomes an opportunity to manage self‐care in everyday life and serves as an important driving force in deepening one's understanding of the significance of self‐care. An active search for new knowledge that is valued in relation to science and expertise makes knowledge understandable and meaningful, which creates a personal experience and understanding. Thus, taking knowledge into one's own hands leads to increased security with the opportunity for participation and personal responsibility.

##### 4.2.3.1 Seeking Knowledge as a Strategy for Control and Understanding

The search for knowledge is based on personal needs and questions, which may mean that the information comes from different sources. Understanding is based on personal experience and comparison with the situations of others. Seeking knowledge is a way to create control and understand the meaning of self‐care.

One participant illustrated this while describing dialogue with their doctor about walking barefoot: ‘I do get a bit of a scolding from my doctor, because I usually want to walk barefoot indoors and sometimes outside. But I'm a bit contrary that way; I like walking barefoot … I walk barefoot indoors, but I never go outside without shoes, I say, although I actually do [laughs] … I think it matters a lot that the feet stay soft, because then they heal faster. If they're dry and cracked, it's more difficult’ (D7).

Personal experience and reasoning interact with professional advice and influence how learning about self‐care of diabetic feet is put into practice. The search for knowledge can also be initiated by experiences of the difficult consequences of the disease within the family. There is an awareness that the ability to access, interpret and understand knowledge varies.

##### 4.2.3.2 Understanding as a Prerequisite for Motivation

Understanding why an action is important is crucial; it makes self‐care more meaningful and provides a sense of personal responsibility. However, when explanations are lacking or contradictory, doubt arises. As one participant expressed: ‘I have to understand … I also find it very hard to stay motivated if I don't understand what I'm supposed to do … I think that's very common. I think it's almost universal that understanding why you do something makes you motivated – and that understanding itself is a driving force’ (D8).

When experts provide clear explanations, it is easier to understand why specific actions are important and they are more likely to be followed. Conversely, a lack of comprehensibility can lead to selective adherence, such as moisturising less frequently if the benefit is not perceived as clear.

Understanding why something is important creates a driving force, an increased motivation. This understanding emphasises the seriousness and importance of self‐care. Dialogue with healthcare professionals is perceived as central to achieving this understanding, but it requires time, responsiveness and mutual engagement. When persons are allowed to ask questions and receive answers, their knowledge and confidence in their own abilities grows.

##### 4.2.3.3 Trust in Science and a Critical Attitude to New Knowledge

Participants expressed a strong degree of trust in scientifically grounded knowledge, alongside an emphasis on maintaining a critical attitude toward different types of information and treatment methods. Trust is built when professionals demonstrate deep knowledge and can explain connections in an understandable way. Conversely, scepticism arises toward anyone who appears overly confident without possessing real expertise. As one participant put it, there is ‘respect for persons with knowledge … and then there are all these quacks who think they know best and make the most noise’ (D6).

Confidence develops when professionals or others communicate well‐founded knowledge, while it becomes difficult to trust those who claim to know but lack experience. Participants expressed trust in scientific knowledge by actively seeking medical information from research articles and relying more on scientific sources than on alternative treatments. Alternative methods were presented with grand promises, but instead provoked scepticism and mistrust, especially when such actors seemed to exploit person's goodwill and vulnerability.

## 5 Discussion

The discussion section integrates comprehensive understanding according to Lindseth and Norberg (2004).

### 5.1 Comprehensive Understanding

The comprehensive understanding of the findings can be highlighted because learning about foot self‐care for persons living with diabetes takes place within a social landscape where learning is shaped and supported both within and beyond the healthcare encounter. Learning occurs through a gradually deepened understanding and is not always linear. The sense of being in one's feet brings insight into the significance of the feet, and self‐care ability develops in an interspace where opportunities, obstacles and bodily symptoms serve as driving forces for action. Taking responsibility for one's own learning is grounded in trust in science, interpreted through one's own understanding and conditions, and approached with a critical mindset.

Van Manen's (1997) theory of lifeworld provides the basis for a discussion on how this learning process can be understood in relation to the aim of the study. The body, as a lived body, does not appear as a neutral object but as an active agent in learning. Through the lifeworld perspective, learning can be seen as a process of ‘finding one's feet’, where the person gradually orients himself or herself and finds meaning within a changed bodily and existential landscape. In this movement, the person learns to navigate vulnerability, loss of control and slowly reorient themselves toward new understanding. Thus, finding one's feet becomes both a bodily and existential act, a way of re‐establishing balance in everyday life. Learning about self‐care is reinforced by experiencing pain, discomfort or fragility in the feet. These feelings bring awareness to the limits and needs of the lived body, and direct attention to what was previously taken for granted. Through bodily awareness such as pain and anxiety, the person begins to reinterpret the meaning of care and seek new knowledge. This aligns with Berglund's (2014) description of turning points in learning among persons with long‐term illness, where the realisation of the illness's impact on life often marks an existential shift. This means that learning is not merely a cognitive process but a deepened understanding in which reflection and dialogue play a decisive role. This also reflects Björk et al.'s (2025) argument that learning begins in bodily experience and as a driving force for learning. It appears that once the body is taken for granted, it becomes a stranger that must be re‐understood. Getting to know your body, its limitations and possibilities is central for foot self‐care.

Time, as lived time (Van Manen 1997), becomes evident as participants move between experiences of the past, present and imagined future. Our results showed that learning about foot self‐care in diabetes is shaped within a social landscape where relationships, support and access to care interact with the person's lived experience, colouring their present understanding and shaping their expectations of the future. However, this social landscape can also be understood as an autonomous space, a room of its own that exists beyond what healthcare can or should not fully control or organise. Within this space, learning unfolds through daily interactions, shared experiences and informal exchanges that extend far beyond the clinical encounter. Learning can be seen as a process that is constantly in motion. This may be interpreted as meaning that person‐centred learning must recognise that each person carries a unique life story that influences what, when and how learning occurs. A foot ulcer that healed years ago may still affect a person's sense of security and readiness for the future, meaning that the past remains present in current learning. Berglund and Källerwald (2012) described this as a balancing act along the line of life, where the future can feel uncertain and learning often occurs in small steps, sometimes too late, sometimes too slowly, but always in rhythm with life itself. It appears that persons with diabetes learn to care for their feet through an ongoing negotiation between the past (previous habits and mistakes), the present (the concrete self‐care of daily life) and the future (the fear of complications and the hope of avoiding them). Kneck (2015) argued that living with diabetes is a lifelong and changing learning process, where understanding of the body, healthcare professionals' roles and one's own needs evolves over time. Learning often occurs informally in daily life, shaped by previous experiences, current situations and future expectations. This is perceived as implying that time in learning becomes extended, a web of experiences where past, present and imagined future continually intersect. Learning can also be understood as something that grows from within, through the interaction between personal experience and external circumstances (Ödman 2006). The inner dimension involves the body's own experiences, emotions and interpretations, while the outer dimension consists of encounters with healthcare professionals, instructions, social contexts and material conditions. Understanding is created in this interplay; learning is not a transfer of fixed knowledge, but a process in which persons with diabetes test, interpret and reshape understanding in relation to their own life, the illness and others. Taking knowledge into one's own hands signifies a transition from external guidance to embodied understanding. The concept of ‘in‐between’ learning and relationships means that learning takes place in a movement between persons and therefore cannot be reduced to simple knowledge transfer (Deleuze and Guattari 1987). In the encounter between healthcare professionals and the person with diabetes, such an in‐between space arises, where experiential and practical knowledge meet, are negotiated and transformed. This space related to the person with diabetes encompasses more than the clinical encounter. Relationships with family, colleagues and the environment also influence how understanding and patterns of action are shaped over time.

Space as lived space (Van Manen 1997) also holds significance. It became clear that spaces can be experienced as both open and closed. Healthcare environments may feel safe but can also become inaccessible when appointments are infrequent, points of contact are limited, a professional's language feels foreign or when the patient's experiences are diminished in ways that can support but maybe more importantly also hinder learning (Berglund and Källerwald 2012). For many, the home became the open space where care was conducted, often together with a partner. This is interpreted as a shift where learning occurs not only in the formal healthcare setting, but within everyday life, where support, sharing responsibility, observing changes, and providing security and maintaining adequate self‐care are negotiated and formed from the open home sphere rather than the contact with sometimes inaccessible health care. This can be interpreted as meaning that the attentiveness of close others, reminding, observing and sharing emotional burdens, can facilitate important aspects of learning. In this context, reflection emerges as an extension of these human relations—a shared meaning‐making process through which experience develops into understanding. This is perceived as meaning that reflection is not merely a cognitive activity, but an existential movement toward understanding. Through reflection, what was previously only a feeling or routine in self‐care may become conscious insight. Kjellsdotter et al. (2020) and Berglund et al. (2024) emphasised that shared reflection is vital for learning among persons with diabetes, as it creates opportunities to share experiences, test ideas and gain emotional support. This is interpreted as meaning that, within reflective movement, understanding emerges not as a sudden realisation, but as something that matures through dialogue, shared experience and quiet contemplation. Learning becomes a process of interpretation, where new insights reshape understanding of both the illness and one's identity. However, learning also involves an element of action, a shift from being guided to taking initiative. This transition is captured by taking knowledge into your own hands, where the person transforms professional advice into situated, embodied knowledge. Through experimentation, adaptation and reflection, participants described how they developed their own strategies for foot self‐care in daily life.

Berglund and Källerwald (2012) further clarified that learning in chronic illness involves a driving force, to live a normal life. When persons with diabetes engage in foot self‐care, it is rarely the feet themselves that are central, but rather the wish to preserve mobility, social participation and life as before. In this sense, ‘the being of the feet’ also expresses the will to stay connected to the world, to move, to belong and to live fully despite vulnerability. Here, the existential dimensions of Van Manen's (1997) lived space and lived time become apparent, as foot self‐care represents a means of safeguarding future freedom of movement and quality of life, something far more profound than a technical routine. Different learning strategies are described in the findings; some learn by trying and doing, while others learn by reflecting on bodily signals or through dialogue with relatives and healthcare professionals. The strategies can be seen as meaning‐making in the interaction among experience, reflection and action. Learning cannot take place in the form of transfer and must instead be interpreted and understood based on one's own life situation. The person‐centred perspective becomes evident: individuals develop their learning while finding their feet through a unique interplay of bodily experience, support from close others and professional guidance. Within this uniqueness, certain patterns have emerged through the understanding presented in this text: the social landscape of self‐care, the being of the feet and taking knowledge into one's own hands. These patterns shape the process in an intricate and immanent movement of continuing learning.

### 5.2 Limitations

Trustworthiness was ensured through strategies addressing credibility, dependability, confirmability and transferability (Lincoln and Guba 1985). Credibility was supported by narrative interviews conducted in participant‐chosen settings, enabling rich descriptions of lived experiences. However, this study does have certain limitations.

Participants were recruited through an advertisement in a public diabetes magazine and via snowball sampling. This strategy enabled access to participants in their everyday life settings, aligning with the study's lifeworld perspective. This approach positively influenced participation by attracting those willing to share personal reflections. Everyone who was interested in participating was interviewed. Recruitment ceased when no new meanings emerged, indicating saturation.

Although the findings are situated in the Swedish context, they may offer insights into learning about foot self‐care as a lived and relational process.

## 6 Conclusions

The being of the feet, the social landscape of self‐care and taking knowledge into your own hands reveal learning as a lived, relational and embodied process. Through these intertwined dimensions, persons with diabetes continuously ‘find their feet’, not only in terms of maintaining physical health, but in terms of reclaiming a sense of control, connection and meaning within their changed lifeworld. Learning in foot self‐care among persons with diabetes must be understood as more than pedagogical strategies and patient information. Person‐centred learning among persons with diabetes constitutes a central foundation for how learning can be developed collectively in the person's encounter with healthcare professionals. When persons' experiences of self‐care related to their feet are made visible and actively utilised in interactions with healthcare professionals, these experiences can contribute to shared learning within clinical practice. For clinical practice, this implies that healthcare professionals need to more actively integrate persons' experience‐based knowledge into care encounters and learning situations. Future research should investigate how personal learning processes can be translated and shared in ways that promote collective knowledge development within the healthcare team, in which the patient is an important and integral participant.

## Author Contributions

Kristofer Björk: conceptualisation, methodology, formal analysis, writing – original draft. Henrik Eriksson: conceptualisation, methodology, formal analysis, writing – review and editing. Ulla Hellstrand Tang: conceptualisation, formal analysis, writing – review and editing. Susanne Andersson: conceptualisation, methodology, formal analysis, writing – review and editing.

## Funding

This work was conducted in collaboration with and supported by the infrastructure of the Swedish Research School in Integrated Care for Future Teachers (SHIFT CARE), funded by the Swedish Research Council (Vetenskapsrådet) (DNr 2022‐06348).

## Consent

All participants received written and verbal information about the study, were informed that participation was voluntary, and provided written informed consent prior to participation.

## Conflicts of Interest

The authors declare no conflicts of interest.

## Supporting information


**Data S1:** COREQ checklist.

## Data Availability

The datasets generated and analysed during the current study are not publicly available as they form part of an ongoing PhD project. However, the data supporting the findings of this study can be obtained from the corresponding author upon reasonable request.
